# Epidemiology, Disease Course, and Clinical Outcomes of Perianal Fistulas and Fissures Crohn’s Disease: A Nationwide Population-Based Study in Taiwan

**DOI:** 10.1093/crocol/otad035

**Published:** 2023-07-25

**Authors:** Meng-Tzu Weng, Kuan-Lin Lin, Ya-Ling Huang, Chitra Karki, Jin-Liern Hong, Dimitri Bennett, K Arnold Chan, Shu-Chen Wei

**Affiliations:** Department of Medical Research, National Taiwan University Hospital Hsin-Chu Branch, HsinChu, Taiwan; Department of Internal Medicine, National Taiwan University Hospital, National Taiwan University, Taipei, Taiwan; Health Data Research Center, National Taiwan University, Taipei, Taiwan; Health Data Research Center, National Taiwan University, Taipei, Taiwan; Global Evidence and Outcomes, Takeda Development Center Americas, Inc., Cambridge, MA, USA; Global Evidence and Outcomes, Takeda Development Center Americas, Inc., Cambridge, MA, USA; Global Evidence and Outcomes, Takeda Development Center Americas, Inc., Cambridge, MA, USA; Perelman School of Medicine, University of Pennsylvania, Philadelphia, PA, USA; Health Data Research Center, National Taiwan University, Taipei, Taiwan; Department of Internal Medicine, National Taiwan University Hospital, National Taiwan University, Taipei, Taiwan

**Keywords:** Crohn’s disease, epidemiology, perianal disease

## Abstract

**Background:**

Population-based data on the course of perianal disease in East Asian populations with Crohn’s disease (CD) are limited. This study examined the prevalence, clinical course, and compared the outcomes of CD patients with perianal CD (pCD) versus without pCD in Taiwan.

**Methods:**

A nationwide population-based study was implemented from 2000 to 2017 by using the Taiwan National Health Insurance Research Database.

**Results:**

Of 2424 patients with CD, 358 (14.8%) patients with pCD were identified. Most patients with CD and pCD were men (79.3%). The mean age at CD diagnosis was lower in patients with pCD (33.7 years) than in those without pCD (44.9 years). Approximately half the patients with pCD received the pCD diagnosis at least 6 months before receiving a CD diagnosis. Approximately one-third (121/358) of patients with pCD had recurrent fistula; the median recurrence interval was 239 days. Compared with patients without pCD, patients with pCD had higher mean incidences of hospitalization (7.0 vs 3.8, *P* < .01), outpatient visits (13 vs 2.9, *P* < .01), and emergency room visits (10.3 vs 4.4, *P* < .01) over a 15-year period. Although patients with pCD had higher rates of healthcare utilization, their 15-year mortality rate was lower than that of those without pCD (6.1% vs 17.3%, *P* < .01).

**Conclusions:**

The period prevalence of pCD in Taiwanese patients with CD was 14.8%. Although patients with pCD required more intensive care and had greater healthcare utilization, they did not have inferior survival outcomes compared with those without pCD.

**Key Messages**

- Population-based data on the epidemiology and course of pCD in East Asia are limited.
- The period prevalence of pCD in Taiwanese patients with CD was 14.8%, with approximately half of patients with pCD receiving a pCD diagnosis at least 6 months before receiving a diagnosis of CD; approximately one-third experienced disease recurrence.
- Patients with pCD required more intensive care and had more healthcare utilization but did not have inferior survival outcomes compared with patients without pCD.

## Introduction

Crohn’s disease (CD) is a chronic inflammatory bowel disease that can affect any portion of the gastrointestinal tract, from the oral cavity to the anus. Perianal disease is one of the most challenging complications of CD and is difficult to treat.^1^ Perianal CD (pCD) can develop at any time during the course of CD. Studies conducted in Western countries have reported that the cumulative incidence of pCD in patients with CD ranges from 16.9% to 21.0% at 10 years after CD diagnosis and 23.0% to 28.3% at 20 years after CD diagnosis.^2–4^ In South Korea, the cumulative incidence of pCD or perianal abscess was reported to be 40.0% at 5 years after CD diagnosis in a population-based cohort and 62.5% at 20 years after diagnosis in a hospital-based cohort.^5^ A meta-analysis suggested that perianal involvement is more frequent in Asian patients with CD than in patients of European descent with CD.^6^ However, no studies have yet investigated the course of pCD in an East Asian population with CD.

A complex condition, pCD compromises the quality of life of patients with CD and is associated with their increased healthcare utilization.^7–9^ This condition can cause physical and sexual impairment and affect psychological well-being. Fecal incontinence may develop in patients with severe pCD. In addition, pCD is associated with an increased need for surgical intervention^7^ and is a predictor of poor long-term outcomes.^10^ A Korean hospital-based cohort study of patients with CD found that pCD was associated with a higher likelihood of mortality.^11^ CD-related fistulas are more aggressive and generally have a lower treatment success rate compared with sporadic fistulas,^12,13^ and treatment failure or fistula relapse is common. Perianal fistulizing CD is typically managed with a combination of medical and surgical approaches.^14^ Elevated levels of proinflammatory cytokines have been detected in anal fistulas and can cause the epithelial-to-mesenchymal transition^15^ and interfere with wound healing. Management of perianal fistulizing CD requires the optimization of medical therapy to reduce inflammation and achieve mucosal healing. Antibiotics are reserved for patients with anorectal abscesses or systemic signs of infection. Seton drainage may enable effective long-term disease control. Techniques such as endorectal advancement flaps and ligation of the intersphincteric fistula tract may be used to correct anatomical abnormalities in the perineum.^14^

No large epidemiological studies based on East-Asian-population-based data have yet investigated the course of pCD in patients with CD or compared the clinical outcomes of CD in patients with versus without pCD. This epidemiological study evaluated the prevalence, clinical characteristics, disease course, and comorbidities of patients with pCD. Furthermore, using a nationwide database in Taiwan, this study compared the outcomes of patients with pCD with those of patients with CD without pCD.

## Materials and Methods

### Study Population

This retrospective cohort study gathered data from Taiwan’s National Health Insurance Research Database (NHIRD) and the Taiwan Cancer Registry (for colorectal cancer identification). Mortality data were also collected. The study protocol was approved by the Institutional Review Board of our Hospital (case number xxxxxxxxxx). The requirement for informed consent was waived because of the use of deidentified data.

Taiwan’s National Health Insurance (NHI) program was established in 1995, and its population coverage rate has consistently been over 99%; the NHI program covered more than 99% of Taiwan’s 23.6 million residents in 2020. The NHI datasets for research comprise demographic characteristic data, enrollment information, and reimbursement records for medical and pharmacy use. This study employed the diagnosis codes of the *International Classification of Diseases, Ninth Revision, Clinical Modification* (*ICD-9-CM*) for the period up to 2016 and the *International Classification of Diseases, Tenth Revision, Clinical Modification* (*ICD-10-CM*) for the period from 2017 onward.

Patients with CD were operationally defined as fulfilling one of the 2 following criteria: (1) having a diagnosis of CD in the Registry for Catastrophic Illness Patient Database (RCIPD) within the period 2000 to 2017 or (2) having at least 6 inpatient or outpatient claims with a diagnosis of CD (*ICD-9-CM* code: 555.0–9, *ICD-10-CM*: K50.0–50.9) on different days within a 12-month period for 2 nonoverlapping periods and having a prescription for at least one medication related to inflammatory bowel disease (ie, 5-aminosalicyclic acid [5-ASA], oral or injectable systemic steroid, immunomodulator, or biologic) within the period 2000-2017. Patients registered as having a diagnosis of CD in the RCIPD all had clinically confirmed CD because their complete medical history and supporting evidence—such as pathology, imaging, and endoscopy findings—were reviewed by the NHI Administration. We established the second criterion to enroll more possible patients with CD because some patients may not have been registered with the RCIPD. We implemented the analysis by using the *ICD-9* code for Crohn’s disease, 555.x (ie, regional enteritis), and found that the case numbers were exceptionally high (ie, approximately 30 000 inpatients and 260 000 outpatients). We suspect that many patients with enterocolitis may have been miscoded as 555.x and thus were misclassified as having CD. Therefore, relying solely on the ICD coding could have led to inaccurate analysis results for CD. Instead of operationally defining patients with CD by using diagnosis codes, we implemented stringent criteria to minimize the risk of enrolling patients who had received a misdiagnosis of CD. This study ensured that patients had at least 6 inpatient or outpatient claims with a diagnosis of CD on different days within a 12-month period for 2 nonoverlapping periods and had a prescription for 5-ASA, a systemic steroid (oral, injectable, or enema), or an immunomodulator. This definition is consistent with the clinical management of CD in Taiwan; patients with active CD make at least 6 annual outpatient clinic visits. In Taiwan, when a patient has an active disease, doctors generally follow up with the patient at regular intervals ranging from days to weeks; in the case of remission, the longest interval for medication refill is 3 months.

**Figure 1.**
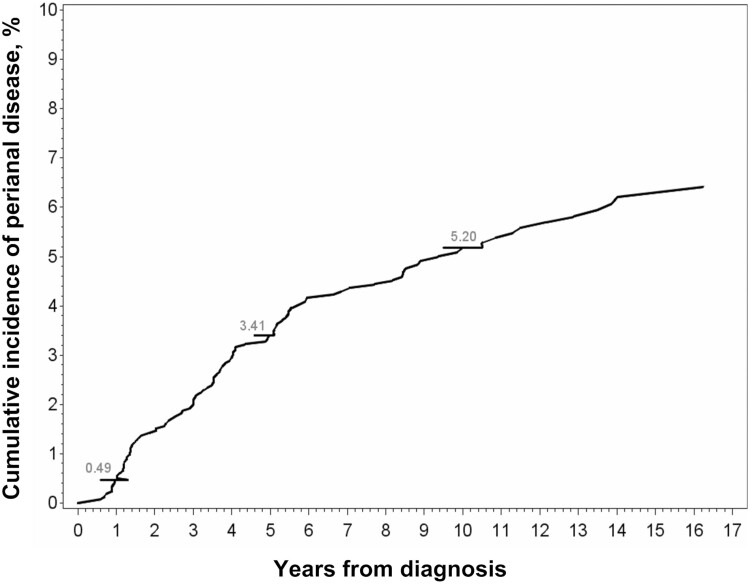
Cumulative incidence of perianal disease in 2424 patients with Crohn’s disease.

A pCD episode was operationally defined as having one or more consecutive healthcare encounter(s) in which a diagnosis was made of perianal fistula (PAF) or anal fissure (*ICD-9-CM*: 565.0 and 565.1; *ICD-10-CM*: K60.0–5) and there was use of systemic antibiotics (ie, metronidazole or ciprofloxacin) or a performance of a surgical procedure (ie, fistulectomy, management of perianal lesion, or management of fistula; corresponding codes are provided in Table S1) within a span of 60 days (Figure 1). Remission of pCD was defined as the absence of additional outpatient visits with a pCD code within the 60 days after treatment with antibiotics or surgery for an episode of pCD. An episode of recurrent pCD was defined as pCD diagnosis code(s) combined with antibiotic use (ie, metronidazole or ciprofloxacin) or surgical procedure beyond the 60 days after the end of the prior pCD episode.

Patients with CD were categorized into 1 of 3 groups on the basis of the apparent temporal sequence between the first CD diagnosis and the first pCD diagnosis: (1) pCD before CD (early onset pCD; pCD diagnosis > 6 months before the CD diagnosis); (2) pCD diagnosis coinciding with CD diagnosis (pCD diagnosis within the 6 months before or after the CD diagnosis); or (3) pCD after CD (pCD diagnosis > 6 months after the CD diagnosis).

We defined comorbidities of interest by using *ICD-9-CM* and *ICD-10-CM* codes; these comorbidities were hypertension, diabetes, chronic kidney disease, coronary artery disease, and liver disease (Table S2). The time window for defining comorbidities extended to any time prior to the first diagnosis of CD or pCD. Patients with CD were operationally defined as having hypertension, diabetes, chronic kidney disease, coronary artery disease, or liver disease if the outpatient diagnosis for any of these conditions occurred at least 3 times on different days or if there was at least one hospital discharge diagnosis of the condition within the 3 years before the first CD diagnosis. Relevant NHI codes are provided in Table S3.

### Outcomes of Interest

The outcomes of interest were surgical procedures for CD complications, the frequency of hospitalization, visits to outpatient departments, visits to emergency rooms, incidence of colorectal cancer (based on data from the Taiwan Cancer Registry), and all-cause mortality (based on data from the mortality database). The incidences of hospitalizations, outpatient visits, and ER visits were restricted to those patients with diagnostic codes related to CD or pCD. For patients with CD without pCD, follow-up began on the date of first CD diagnosis. For patients with CD and pCD, follow-up began at the time of first diagnosis of CD or pCD. The follow-up continued until the date of death or December 31, 2017, whichever occurred first. The outcome of colorectal cancer was evaluated through to the end of 2016 because of the limited data available on the Taiwan Cancer Registry.

### Statistical Analysis

Descriptive data are expressed as counts, percentages, means with standard deviations (SDs), or medians with interquartile ranges (IQRs) as appropriate. For comparisons of groups, this study employed the chi-square test for categorical variables and Student’s *t*-test for continuous variables. Various follow-up periods were evaluated, and the results for a 3-year follow-up are presented. Statistical significance was indicated at *P* < .05. The Kaplan–Meier method was employed to evaluate the cumulative incidences of CD and pCD and to generate the corresponding recurrence curves. The prevalence of pCD was calculated using the number of patients with pCD divided by the number of patients with CD at a certain time point; period prevalence was calculated using the number of patients with pCD divided by the total number of patients with CD during a specific period. To calculate the duration of pCD remission, this study assigned each pCD episode a start date and an end date, and the interval between 2 pCD episodes was measured accordingly.

## Results

### Baseline Characteristics of Patients

Table 1 presents the baseline characteristics of patients with CD. We identified 2424 patients with CD in Taiwan between 2000 and 2017 (1230 patients identified using the manual method who were registered in the RCIPD and 1194 patients identified using the manual method who were not registered in the RCIPD). Of these patients, 358 (14.8%) had a diagnosis of pCD (Table 1). Most patients with CD were male (63.2%), and a higher proportion of patients with CD and pCD were men (79.3%) than were women. The patients with CD and pCD had a lower mean age (33.7 years, SD: 14.9 years) than those without pCD (44.9 years, SD: 21.8 years). The mean age at onset of pCD was 33.1 years (SD: 15.4 years).

**Table 1.** Baseline Characteristics of 2424 Patients With Crohn’s Disease in Taiwan.

	With perianal disease	Without perianal disease	*P*-value
	*N* (%)	*N* (%)	
Total	358 (100.0)	2066 (100.0)	
Sex			<.01
Male	284 (79.3)	1248 (60.4)	
Female	74 (20.7)	818 (39.6)	
Age at diagnosis of Crohn’s disease			
Mean (SD)	33.7 (14.9)	44.9 (21.8)	<.01
Age at diagnosis of perianal disease			
Mean (SD)	33.1 (15.4)		
Comorbidity during a three-year baseline period			
Hypertension	23 (6.4)	359 (17.4)	<.01
Diabetes	11 (3.1)	182 (8.8)	<.01
Chronic kidney disease	3 (0.8)	31 (1.5)	.47
Coronary artery disease	8 (2.2)	143 (6.9)	<.01
Liver disease	42 (11.7)	232 (11.2)	.79

During the 3 years before the patients’ cohort entry date, the incidence of the following underlying comorbidities was lower in the group of patients with CD and pCD than in the group of patients with CD without pCD: hypertension (6.4% vs 17.4%, *P* < .01), diabetes (3.1% vs 8.8%, *P* < .01), and coronary artery disease (2.2% vs 6.9%, *P* < .01).

Patients with CD and pCD were more likely to receive 5-ASA (93.9% vs 68.3%, *P* < .01), a steroid (93.9% vs 86.9%, *P < *.01), azathioprine (61.7% vs 30.4%, *P* < .01), a tumor necrosis factor (TNF)-alpha inhibitor (44.4% vs 15.3%, *P* < 0.01), vedolizumab (2.0% vs 0.1%, *P* < .01), and an antibiotic (79.9% vs 54.1%, *P* < .01) compared with the patients with CD without pCD (Table 2).

**Table 2.** Drug Use in 2424 Patients with Crohn’s Disease in Taiwan.

	With perianal disease	Without perianal disease	*P*-value
	*N* (%)	*N* (%)	
Total	358 (100.0)	2066 (100.0)	
Medication			
5-ASA	336 (93.9)	1412 (68.3)	<.01
Steroid	336 (93.9)	1796 (86.9)	<.01
Azathioprine	221 (61.7)	629 (30.4)	<.01
Anti-TNF-alpha	159 (44.4)	316 (15.3)	<.01
Vedolizumab	7 (2.0)	3 (0.1)	<.01
Antibiotics	286 (79.9)	1,117 (54.1)	<.01
Metronidazole	266 (74.3)	907 (43.9)	<.01
Ciprofloxacin	167 (46.6)	572 (27.7)	<.01

5-ASA: 5-aminosalicyclic acid; TNF: tumor necrosis factor.

### Prevalence, Time Sequence, and Course of pCD

Of the 358 patients with at least one episode of pCD, 170 (47.5%) had pCD before receiving a CD diagnosis, with the median duration from the first pCD episode to CD diagnosis being 1237.5 days (approximately 3.4 years). For 74 (20.7%) patients, pCD was diagnosed within 6 months of CD diagnosis, with the median duration from CD to pCD diagnosis being 44 days. For 114 (31.8%) patients with CD, pCD was diagnosed a median of 1350.5 days after the CD diagnosis was made (approximately 3.7 years; Table 3). Excluding patients who received a pCD diagnosis before a CD diagnosis and those who received a pCD diagnosis at the time of a CD diagnosis, the incidence of pCD in the 2180 patients with CD who did not have pCD at the time of CD diagnosis was 0.5% at 1 year after CD diagnosis, 3.7% at 5 years, and 5.8% at 10 years (Figure 1). For all 2424 patients with CD, the cumulative incidence of pCD was 10.1% at 1 year, 12.9% at 5 years, and 14.8% at 10 years.

**Table 3.** Temporal Sequence Between First Diagnosis of Crohn’s Disease and First Diagnosis of Perianal Disease in 358 Patients in Taiwan.

Diagnosis of perianal disease	*N* = 358	Days between first Crohn’s disease diagnosis and first diagnosis of perianal disease	
		Mean (SD)	Median (IQR)
More than 6 months before diagnosis of Crohn’s disease	170	1576 (1,188)	1237.5 (707-2,237)
Within 6 months before or after diagnosis of Crohn’s disease	74	57 (51)	44 (9-95)
More than 6 months after diagnosis of Crohn’s disease	114	1762 (1394)	1350.5 (606-2417)

IQR: interquartile range.

**Figure 2.**
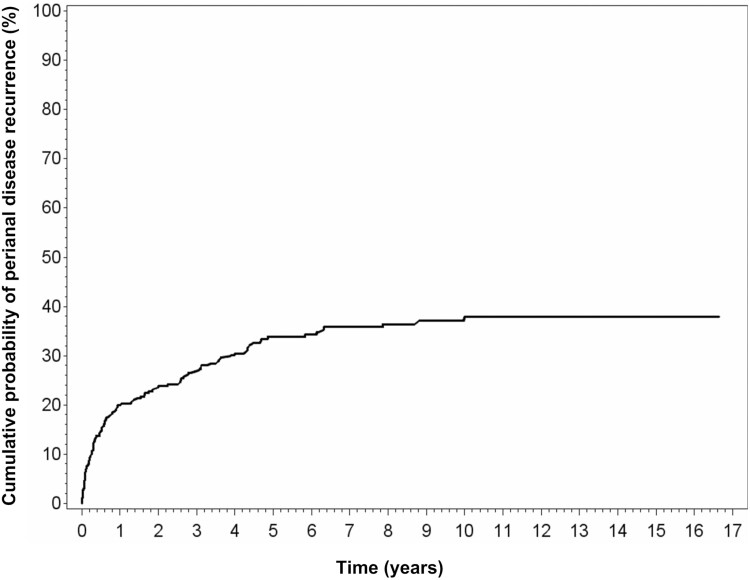
Cumulative probability of first episode of perianal disease recurrence in 358 patients with Crohn’s disease.

Of the 358 patients with CD and pCD, 121 (33.8%) had recurrent pCD during the follow-up period. The median duration of the first pCD episode was 116 days, and the median duration of the second, third, fourth, and fifth episodes ranged between 130 and 148 days (Table S4). The median durations of the first, second, and third pCD remissions were 268, 583, and 135 days, respectively (Table 4). The cumulative probability of the first episode of pCD recurrence was 19.4% at 1 year, 34.1% at 5 years, and 38.4% at 10 years when considering all the patients with pCD (Figure 2).

**Table 4.** Interval Duration Between Episodes of Perianal Disease in 358 Patients With Crohn’s Disease.

Recurrence interval	Number of episodes of second or subsequent perianal disease	Days between intervals
		Median (IQR)
Total	207	239 (68-902)
First recurrence	121	268 (77-1012)
Second recurrence	45	583 (66-1241)
Third recurrence	19	135 (45-260)
Forth recurrence	10	258 (143-521)
Fifth recurrence	6	224 (31-437)
Six (and more) recurrence	6	110 (45-136)

IQR: interquartile range.

We also implemented an analysis of patients with PAF who were identified through the RCIPD (Table 5). Of the 256 patients, 119 (46.5%) received their pCD diagnosis before receiving a CD diagnosis. The results were similar for patients from both the RCIPD and NHI datasets, with 170 out of 385 patients (47.5%) receiving a pCD diagnosis before receiving a CD diagnosis.

### Outcomes of Patients With CD With and Without pCD

The 15-year follow-up data for patients with CD with and without pCD are presented in Table 5. Patients with CD and pCD were more likely to undergo surgical intervention (1.4 vs 1.2, *P* < .01), be hospitalized (7.0 vs 3.8, *P* < .01), and make outpatient department visits (13.0 vs 2.9, *P* < .01) and ER visits (10.3 vs 4.4, *P* < .01) than were those without pCD. The 15-year mortality rate was lower in patients with pCD than in those without pCD (6.1% vs 17.3%, *P* < .01; Table 5). A total of 23 of the patients with CD received a diagnosis of colorectal cancer within the 15-year follow-up; the incidence of colorectal cancer did not differ significantly between the 2 groups.

**Table 5.** Outcomes of 2424 Patients With Crohn’s Disease With or Without Perianal Disease During 15-Year Follow-Up.

	With perianal disease *N* = 358		Without per-anal disease *N* = 2066		*P*-value
	*N*	Episodes Mean (SD)	*N*	Episodes Mean (SD)	
Surgical intervention	153	1.4 (0.8)	378	1.2 (0.5)	<.01
Hospitalization	356	7.0 (6.7)	907	3.8 (4.6)	<.01
Outpatient visits	358	13.0 (11.3)	2017	2.9 (2.7)	<.01
Emergency visit	332	10.3 (20.2)	642	4.4 (7.3)	<.01
	N	(%)	N	(%)	
Colorectal cancer	4	1.1	19	0.9	.77
Death	22	6.1	358	17.3 %	<.01

### Time of pCD Diagnosis

The 170 patients with pCD who developed perianal disease at least 6 months before receiving a CD diagnosis (ie, early onset pCD) had lower mean numbers of hospitalizations (2.5 vs 3.7, *P* < .01), outpatient visits (18.5 vs 23.1, *P *= .02), and ER visits (2.9 vs 4.3, *P* < .01) compared with the 188 (74 and 114, Table 3) patients who were diagnosed with perianal disease less than 6 months before or after the time of CD diagnosis (pCD on/after CD diagnosis; Table S6). Further analysis revealed that more patients in the early onset pCD group than in the pCD on/after CD diagnosis group received an early biologic during the 3-year follow-up (24.1% vs 11.7%, *P* < .01).

We also implemented a subanalysis of patients who received a diagnosis of PAF; the results are presented in Tables S7–S11. Patients with PAF were generally younger than those without PAF (33 vs 44.8 years). Additionally, patients with CD and PAF were less likely to have underlying comorbidities, such as hypertension, diabetes, and coronary artery disease (Table S7). Patients with PAF received more medication, such as 5-ASA, steroids, azathioprine, anti-TNF agents, vedolizumab, and antibiotics (*P* < .01; Table S8). Of all the patients with PAF, 206 (66.8%) had PAF either before or at the time of CD diagnosis (Table S9). The durations between episodes of PAF are presented in Table S10. The rates of surgical intervention, hospitalization, outpatient visits, and ER visits (Table S11) were higher in the subgroup of patients with PAF than in those without PAF. These results are consistent with the findings for patients with a diagnosis of perianal disease (Tables 1–4).

## Discussion

To the best of our knowledge, this population-based epidemiological study of residents in Taiwan is the first to evaluate the prevalence and course of pCD as well as the comorbidities of patients with CD and pCD and to compare the outcomes of patients with CD with versus without pCD. One crucial finding is that the period prevalence of pCD in patients with CD in Taiwan was 14.8%. Nearly half the patients with pCD had received the diagnosis at least 6 months before receiving the initial diagnosis of CD. Another critical finding is that one-third of patients with pCD experienced recurrence of the disease. Our results revealed that these patients developed pCD at a median age of 29 years, consistent with the results of relevant studies asserting that young age is a risk factor for perianal disease.^16–18^ Although patients with pCD had a higher level of healthcare utilization than did patients without pCD, patients with pCD did not have inferior survival outcomes.

The age at onset of CD was higher in our study population than in other Asian populations. The median ages at onset of CD in Korean and Hong Kong populations were 22.8 and 28.6 years, respectively.^5,17^ This discrepancy is related to our strict diagnostic criteria, which may have resulted in delayed diagnoses. Results from our previous study, in which data from the RCIPD were employed, revealed a median age at onset of CD of 35 years.^19^ Patients listed in the RCIPD accounted for approximately one-third of all patients with CD enrolled in this study.

One study reported that the incidence of pCD in Asia ranges from 30.3% to 58.8%,^20^ but our results revealed that the incidence of pCD was 14.8%. Several reasons may exist for this lower prevalence of pCD in our study. First, the definition of pCD in our study differs from that used in previously published studies. Most relevant studies have identified patients with perianal diseases (eg, perianal abscess, skin tags, and hemorrhoids), but we only identified patients with PAF or anal fissures. Second, the data sources in this study differ from those in relevant studies. This study employed population-based data, whereas most other studies have employed patient data from referral centers. Third, this study used operational definitions that incorporated information on diagnostic codes, drug use, and surgical procedures from health insurance claims. Our analysis may have omitted patients with pCD who were only treated with a biologic agent or immunosuppressive agent but without antibiotics and/or surgery, although we believe that the number of patients in this group would be low in our cohort. Finally, the widespread use of immunosuppressive agents and anti-TNF agents in recent decades may have led to a decrease in the incidence of pCD in patients with CD. Park et al reported a 10-year rate of perianal or rectovaginal fistula of 12% in patients who received a diagnosis of CD after 1998 and a rate of 24% in those receiving a diagnosis before 1998.^4^ Song et al. found that the cumulative incidence of pCD was significantly lower in or after 2005 than that before 2005.^5^ Our results revealed that patients with pCD were treated with 5-ASA, steroids, immunomodulators, and biologics more often than those without perianal pCD; these results align with those of hospital-based studies conducted in Korea^10,21^ and a nationwide cohort study from Denmark.^22^ Although 5-ASA is not recommended for treating patients with CD, a high proportion of patients with CD (82.1%) were treated with 5-ASA in Taiwan, as reported in our previous study^19^; this finding could be related to NHI reimbursement criteria.

Notably, of the 358 patients with pCD who were identified in this study, 47.5% developed pCD before receiving a CD diagnosis, and 20.7% developed pCD at approximately the same time as their first CD diagnosis. In other Asian regions, 30% of patients in a territory-wide study conducted in Hong Kong^17^ and 70.9% of patients in a nationwide study in Korea presented with perianal disease before receiving a CD diagnosis.^5^ These findings suggest that pCD may be an early sign of CD in East Asian populations. In a nationwide Danish study, 50% of patients with pCD developed perianal disease before receiving a CD diagnosis; 11% of patients presented with perianal disease at the time of CD diagnosis; and the remaining 39% developed perianal disease after CD diagnosis^22^; these findings are consistent with the aforementioned findings. The high proportion of pCD cases being identified before CD could be attributable to delayed diagnosis of CD or be a result of the natural progression of CD. Studies have revealed that the interval between the first symptoms of CD and the diagnosis of CD ranges from 5 to 9 months in Western countries^23–26^ and from 6.2 to 83 months in Asian countries.^27–29^ The results of the present study revealed a median interval period of approximately 3.4 years from pCD to CD. The median duration for patients with CD who later received a diagnosis of pCD was 3.7 years.

Notably, approximately one-third of patients with CD and pCD in our study experienced at least one recurrence of pCD. One meta-analysis reported fistula recurrence rates ranging from 0% to 83.3% in a seton drainage group and from 8% to 40.9% in an anti-TNF treatment group.^30^ In a retrospective cohort from France, the cumulative probability of first fistula recurrence was 16.6% and 40.1% at 1 and 5 years, respectively, for patients treated with infliximab.^31^ Given the standard treatment in Taiwan, the observed recurrence rate is similar to that in relevant studies. In addition, the median interval to the first recurrent episode was approximately 9 months (IQR: 77 days to 2.8 years), and the median duration of the first or subsequent pCD episode ranged from 116 to 270 days.

The present results revealed higher incidences of hospitalizations, outpatient visits, and ER visits in patients with CD and pCD than in patients with CD without pCD. pCD is an aggressive clinical phenotype of CD, and a higher rate of surgical procedures was observed in a group of patients with pCD in a hospital-based study conducted in Korea^21^ and in a population-based study from Denmark.^32^ Dias and colleagues reported a significantly high probability of events in patients with pCD, including surgery, hospitalizations, steroid dependency, use of immunosuppressants or anti-TNF agents, or new events such as stenosis, penetrating disease.^33^ Studies have established the effectiveness of anti-TNF agents (eg, infliximab and adalimumab) for treating perianal fistulizing CD,^34–38^ and high infliximab trough levels during the induction or maintenance phases of treatment are associated with better fistula response.^39^ More frequent outpatient visits may be related to early intensive treatment in patients with pCD. In addition, our results indicated a lower mortality rate in patients with CD and pCD than in patients without pCD. Possible reasons for this finding include age and management of CD. Patients without pCD in this study were older and had more comorbidities (eg, hypertension, diabetes, and coronary artery disease; Table 1); patients with pCD received more intensive treatment and monitoring in the form of medical treatment and outpatient visits.

We discovered that lower incidences of hospitalizations, outpatient visits, ER visits, and mortality in patients with early onset pCD than in patients with non-early onset pCD. This finding may be related to increased physician awareness of the development of CD in patients with pCD and early treatment with biologics. A Danish study evaluated the course of pCD and asserted that patients with pCD were more likely to undergo major abdominal surgery than were patients without pCD.^40^ No relevant studies from Asian countries have examined this topic.

This study has several notable strengths. First, this study is the first nationwide study conducted in East Asia to comprehensively assess the course of pCD in patients with CD with respect to pCD frequency, duration, and recurrence interval. This was achieved by gathering data from Taiwan’s NHIRD. Second, our study also evaluated potential clinical outcomes associated with the time of onset of pCD. Third, we employed a robust methodology to implement all analyses. However, this study also has several limitations that should be noted. Although the retrospective study period was long, the follow-up time for patients who received a diagnosis of CD or pCD in a few years before 2017 may have been insufficient in terms of their subsequent conditions; this may have resulted in potentially inaccurate estimates of pCD prevalence. Certain clinical information was not available in health insurance claims, including disease severity, involved locations, the type of fistula (ie, simple or complex), endoscopy reports, and operation findings. Because we were unable to obtain detailed clinical records, the definition of an episode of pCD recurrence was based on the presence of pCD diagnosis code(s) combined with treatment involving antibiotics or surgical procedures beyond the 60 days after the end of the prior pCD episode. However, this definition may have led to an overestimation of the actual number of pCD cases. This study did not account for the emigration of patients. However, since the net migration rate in Taiwan was around 1-2 per 1000 population,^41^ therefore, emigration is unlikely to significantly affect the current results. The small number of patients with CD and pCD who were only treated with anti-TNF agents was not identified because our operational definition of pCD involved only treatment with antibiotics or specific surgical procedures. Furthermore, analyses comparing outcomes for patients with versus without pCD were not adjusted for potential confounders. Thus, the observed associations between pCD and outcomes were likely affected by confounding bias.

## Conclusion

The prevalence of pCD in Taiwanese patients with CD was 14.8%. The patients who developed pCD had a higher level of healthcare utilization. With respect to the onset of pCD, 47.5% of patients with pCD experienced early onset pCD, and those with early onset pCD were hospitalized less frequently, made fewer outpatient and ER visits, and had a lower likelihood of mortality than patients diagnosed with pCD at the time of CD diagnosis or within the 3 years of follow-up. The fistula recurrence rate was 33.8%, and the median recurrence interval was 239 days. The findings imply that increased awareness of pCD in East Asia is contributing to the early diagnosis of CD and improved clinical outcomes.

## Supplementary Material

otad035_suppl_Supplementary_Materials

## Data Availability

The data from Taiwan’s National Health Insurance Research Database (NHIRD) and Catastrophic Illness Patient Database (RCIPD) will be shared on reasonable request to the National Health Research Institutes (NHRI).

## References

[1] Rackovsky O , Hirten R, Ungaro R, Colombel J-F. Clinical updates on perianal fistulas in Crohn’s disease. Expert Rev Gastroenterol Hepatol. 2018;12(6):597-605. 29792734 10.1080/17474124.2018.1480936

[2] Schwartz DA , Loftus EV, Jr, Tremaine WJ, et al. The natural history of fistulizing Crohn’s disease in Olmsted County, Minnesota. Gastroenterology. 2002;122(4):875-880. 11910338 10.1053/gast.2002.32362

[3] Eglinton TW , Barclay ML, Gearry RB, Frizelle FA. The spectrum of perianal Crohn’s disease in a population-based cohort. Dis Colon Rectum. 2012;55(7):773-777. 22706129 10.1097/DCR.0b013e31825228b0

[4] Park SH , Aniwan S, Scott Harmsen W, et al. Update on the natural course of fistulizing perianal Crohn’s disease in a population-based cohort. Inflamm Bowel Dis. 2019;25(6):1054-1060. 30346531 10.1093/ibd/izy329 PMC6505440

[5] Song EM , Lee HS, Kim YJ, et al. Incidence and outcomes of perianal disease in an Asian population with Crohn’s disease: a nationwide population-based study. Dig Dis Sci. 2020;65(4):1189-1196. 31485994 10.1007/s10620-019-05819-9

[6] Shi HY , Levy AN, Trivedi HD, Chan FKL, Ng SC, Ananthakrishnan AN. Ethnicity influences phenotype and outcomes in inflammatory bowel disease: a systematic review and meta-analysis of population-based studies. Clin Gastroenterol Hepatol. 2018;16(2):190-197.e11. 28603049 10.1016/j.cgh.2017.05.047 PMC5722715

[7] Zwintscher NP , Shah PM, Argawal A, et al. The impact of perianal disease in young patients with inflammatory bowel disease. Int J Colorectal Dis. 2015;30(9):1275-1279. 25994782 10.1007/s00384-015-2251-5

[8] Cohen RD , Waters HC, Tang B, Rahman MI. Effects of fistula on healthcare costs and utilization for patients with Crohn’s disease treated in a managed care environment. Inflamm Bowel Dis. 2008;14(12):1707-1714. 18618630 10.1002/ibd.20530

[9] Mahadev S , Young JM, Selby W, Solomon MJ. Quality of life in perianal Crohn’s disease: what do patients consider important? Dis Colon Rectum. 2011;54(5):579-585. 21471759 10.1007/DCR.0b013e3182099d9e

[10] Chun J , Im JP, Kim JW, et al. Association of perianal fistulas with clinical features and prognosis of Crohn’s disease in Korea: results from the CONNECT study. Gut Liver. 2018;12(5):544-554. 30037171 10.5009/gnl18157 PMC6143449

[11] Lee HS , Choe J, Kim SO, et al. Overall and cause-specific mortality in Korean patients with inflammatory bowel disease: a hospital-based cohort study. J Gastroenterol Hepatol. 2017;32(4):782-788. 27637573 10.1111/jgh.13596

[12] Yang BL , Chen YG, Gu YF, et al. Long-term outcome of infliximab combined with surgery for perianal fistulizing Crohn’s disease. World J Gastroenterol. 2015;21(8):2475-2482. 25741157 10.3748/wjg.v21.i8.2475 PMC4342926

[13] Zalieckas JM. Treatment of perianal Crohn’s disease. Semin Pediatr Surg. 2017;26(6):391-397. 29126509 10.1053/j.sempedsurg.2017.10.009

[14] Gaertner WB , Burgess PL, Davids JS, et al.; Clinical Practice Guidelines Committee of the American Society of Colon and Rectal Surgeons. The American Society of Colon and Rectal Surgeons Clinical Practice Guidelines for the management of anorectal abscess, fistula-in-ano, and rectovaginal fistula. Dis Colon Rectum. 2022;65(8):964-985. 35732009 10.1097/DCR.0000000000002473

[15] Panes J , Rimola J. Perianal fistulizing Crohn’s disease: pathogenesis, diagnosis and therapy. Nat Rev Gastroenterol Hepatol. 2017;14(11):652-664. 28790453 10.1038/nrgastro.2017.104

[16] Cosnes J , Cattan S, Blain A, et al. Long-term evolution of disease behavior of Crohn’s disease. Inflamm Bowel Dis. 2002;8(4):244-250. 12131607 10.1097/00054725-200207000-00002

[17] Mak WY , Mak OS, Lee CK, et al. Significant medical and surgical morbidity in perianal Crohn’s disease: results from a territory-wide study. J Crohns Colitis. 2018;12(12):1392-1398. 30165543 10.1093/ecco-jcc/jjy120

[18] Ingle SB , Loftus EV, Jr. The natural history of perianal Crohn’s disease. Dig Liver Dis. 2007;39(10):963-969. 17720635 10.1016/j.dld.2007.07.154

[19] Weng MT , Tung CC, Chang YT, et al. Trends of medication usage and associated outcomes for Taiwanese patients with inflammatory bowel disease from 2001 to 2015. J Clin Med. 2018;7(11):394. 30373275 10.3390/jcm7110394 PMC6262469

[20] Im JP. Adalimumab or infliximab: which is better for perianal fistula in Crohn’s disease? Intest Res. 2017;15(2):147-148. 28522942 10.5217/ir.2017.15.2.147 PMC5430004

[21] Song EM , Lee HS, Kim YJ, et al. Clinical outcomes and long-term prognosis of perianal Crohn’s disease in an Asian population. J Gastroenterol Hepatol. 2021;36(6):1571-1579. 33091187 10.1111/jgh.15308

[22] Wewer MD , Zhao M, Nordholm-Carstensen A, Weimers P, Seidelin JB, Burisch J. The incidence and disease course of perianal Crohn’s disease: a Danish nationwide cohort study, 1997–2015. J Crohns Colitis. 2021;15(1):5-13. 32582937 10.1093/ecco-jcc/jjaa118

[23] Fiorino G , Danese S. Diagnostic delay in Crohn’s disease: time for red flags. Dig Dis Sci. 2016;61(11):3097-3098. 27638835 10.1007/s10620-016-4298-8

[24] Romberg-Camps MJ , Hesselink-van de Kruijs MA, Schouten LJ, et al. Inflammatory Bowel Disease in South Limburg (the Netherlands) 1991–2002: Incidence, diagnostic delay, and seasonal variations in onset of symptoms. J Crohns Colitis. 2009;3(2):115-124. 21172254 10.1016/j.crohns.2008.12.002

[25] Vavricka SR , Spigaglia SM, Rogler G, et al.; Swiss IBD Cohort Study Group. Systematic evaluation of risk factors for diagnostic delay in inflammatory bowel disease. Inflamm Bowel Dis. 2012;18(3):496-505. 21509908 10.1002/ibd.21719

[26] Nahon S , Lahmek P, Lesgourgues B, et al. Diagnostic delay in a French cohort of Crohn’s disease patients. J Crohns Colitis. 2014;8(9):964-969. 24529604 10.1016/j.crohns.2014.01.023

[27] Lee DW , Koo JS, Choe JW, et al. Diagnostic delay in inflammatory bowel disease increases the risk of intestinal surgery. World J Gastroenterol. 2017;23(35):6474-6481. 29085197 10.3748/wjg.v23.i35.6474 PMC5643273

[28] Moon CM , Jung SA, Kim SE, et al.; CONNECT study group. Clinical factors and disease course related to diagnostic delay in Korean Crohn’s disease patients: results from the CONNECT study. PLoS One. 2015;10(12):e0144390. 26647084 10.1371/journal.pone.0144390 PMC4672933

[29] Li Y , Ren J, Wang G, et al. Diagnostic delay in Crohn’s disease is associated with increased rate of abdominal surgery: a retrospective study in Chinese patients. Dig Liver Dis. 2015;47(7):544-548. 25840874 10.1016/j.dld.2015.03.004

[30] de Groof EJ , Sahami S, Lucas C, Ponsioen CY, Bemelman WA, Buskens CJ. Treatment of perianal fistula in Crohn’s disease: a systematic review and meta-analysis comparing seton drainage and anti-tumour necrosis factor treatment. Colorectal Dis. 2016;18(7):667-675. 26921847 10.1111/codi.13311

[31] Bouguen G , Siproudhis L, Gizard E, et al. Long-term outcome of perianal fistulizing Crohn’s disease treated with infliximab. Clin Gastroenterol Hepatol. 2013;11(8):975-81.e1. 23376316 10.1016/j.cgh.2012.12.042

[32] Zhao M , Lo BZS, Vester-Andersen MK, Vind I, Bendtsen F, Burisch J. A 10-Year follow-up study of the natural history of perianal Crohn’s disease in a Danish population-based inception cohort. Inflamm Bowel Dis. 2019;25(7):1227-1236. 30576474 10.1093/ibd/izy374

[33] Dias CC , Rodrigues PP, Coelho R, et al.; on behalf GEDII. Development and validation of risk matrices for Crohn’s disease outcomes in patients who underwent early therapeutic interventions. J Crohns Colitis. 2017;11(4):445-453. 27683799 10.1093/ecco-jcc/jjw171

[34] Present DH , Rutgeerts P, Targan S, et al. Infliximab for the treatment of fistulas in patients with Crohn’s disease. N Engl J Med. 1999;340(18):1398-1405. 10228190 10.1056/NEJM199905063401804

[35] Rodrigo L , Perez-Pariente JM, Fuentes D, et al. Retreatment and maintenance therapy with infliximab in fistulizing Crohn’s disease. Rev Esp Enferm Dig. 2004;96(8):548-54; 554. 15449986 10.4321/s1130-01082004000800004

[36] Sands BE , Anderson FH, Bernstein CN, et al. Infliximab maintenance therapy for fistulizing Crohn’s disease. N Engl J Med. 2004;350(9):876-885. 14985485 10.1056/NEJMoa030815

[37] Taxonera C , Rodrigo L, Casellas F, et al. Infliximab maintenance therapy is associated with decreases in direct resource use in patients with luminal or fistulizing Crohn’s disease. J Clin Gastroenterol. 2009;43(10):950-956. 19448569 10.1097/MCG.0b013e3181986917

[38] Colombel JF , Schwartz DA, Sandborn WJ, et al. Adalimumab for the treatment of fistulas in patients with Crohn’s disease. Gut. 2009;58(7):940-948. 19201775 10.1136/gut.2008.159251 PMC2689393

[39] Davidov Y , Ungar B, Bar-Yoseph H, et al. Association of induction infliximab levels with clinical response in perianal Crohn’s disease. J Crohns Colitis. 2017;11(5):549-555. 28453755 10.1093/ecco-jcc/jjw182

[40] Wewer MD , Zhao M, Nordholm-Carstensen A, Weimers P, Seidelin JB, Burisch J. The incidence and disease course of perianal Crohn’s disease: a Danish Nationwide Cohort Study, 1997-2015. J Crohns Colitis. 2021;15(1):5-13. 32582937 10.1093/ecco-jcc/jjaa118

[41] Taiwan Net Migration Rate 1950-2023. https://www.macrotrends.net/countries/TWN/taiwan/net-migration.

