# Supplementary material for: Epidemiology, Disease Course, and Clinical Outcomes of Perianal Fistulas and Fissures Crohn’s Disease: A Nationwide Population-Based Study in Taiwan
Source: Crohns Colitis 360. 2023 Jul 25;5(3):otad035. doi: 10.1093/crocol/otad035 (PMC10368329; doi:10.1093/crocol/otad035)
Supplement: otad035_suppl_Supplementary_Materials [file otad035_suppl_supplementary_materials.docx]

**SUPPLEMENTARY MATERIAL FILE**

**Supplementary Figure 1. Operational definition for perianal Crohn’s disease**

**
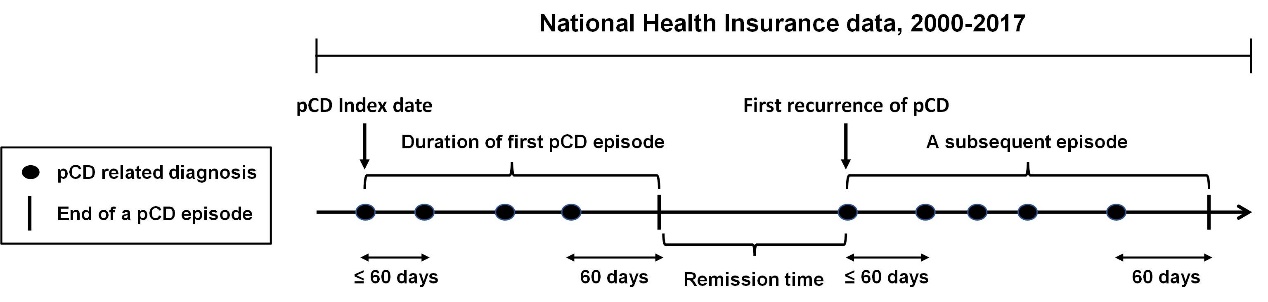
**

**Supplementary Table S1. Surgical procedure codes in Taiwan National Health Insurance (NHI) that may be related to management of perianal Crohn’s disease**

| **NHI Code** | **Description** |
| --- | --- |
| **1. Fistulotomy / Fistulectomy** | |
| 49007C | Fistula curretage |
| 74401C | Fistulotomy or fistulectomy,simple, subcutaneous |
| 74403C | Fissurectomy or ulcerectomy, anal |
| 74411C | Anal fistulectomy or fistulotomy with hemorrhoidectomy |
| 74420C | Fistulotomy or fistulectomy,complicated, subcutaneous |
| 92024B | Fistulectomy |
| **2. Removal / management perianal lesion** | |
| 74215B | Closure fistula,reco-vesical |
| 79601C | Incision and drainage of perineal abscess (Non-obstetric) |
| 80020B | Rectovaginal fistula closure |
| 80021B | Primary recto-vaginal fistula repair |
| 80032B | Recurrent recto-vaginal fistula repair |
| 49010C | Periproctal abscess drainage |
| 74201C | Incision and drainage for periproctal abscess |
| **3. Management of other fistula** | |
| 73026B | Closure of colon fistula-colocutaneous |
| 73027B | Closure of colon fistula-gastroclic without gastrectomy |
| 73028B | Closure of colon fistula-gastroclic with gastrectomy |
| 73029B | Closure of colon fistula-fistula of colon with other organs or complicated |
| 74003B | Closure of appendiceal fistula |
| 77020B | Closure fistula, ureterocutaneous |
| 77021B | Closuer fistula, ureterovisceral |
| 78020B | Closure fistula, vesicouterine with or without hysterectomy |
| 78217B | Repair of urethral cutanous fistula |
| 80022B | Urethral vaginal fistula repair |
| 80023B | Vesico vaginal fistula repair |
| 78218B | Urethral fistulectomy(posterior) |
| 78220B | Urethral fistulectomy(anterior) |

**Supplementary Table S2. International Classification of Disease (ICD) Clinical Modification (CM) codes for selected comorbidity**

| Comorbidity |  | Diagnosis code |
| --- | --- | --- |
| Hypertension | ICD-9-CM | 401.x |
|  | ICD-10-CM | I10.x |
| Diabetes | ICD-9-CM | 250.x |
|  | ICD-10-CM | E08-E11.x, E13.x |
| Chronic kidney disease | ICD-9-CM | 585.x |
|  | ICD-10-CM | N18.x |
| Coronary artery disease | ICD-9-CM | 414.x |
|  | ICD-10-CM | I25.x |
| Liver disease | ICD-9-CM | 070.x, 571.x |
|  | ICD-10-CM | B15-B19.x, K70.x, K73-75.x |
| Colorectal cancer | ICD-9-CM | 153-154.x |
|  | ICD-10-CM | C18-21.x |

**Supplementary Table S3. Surgical procedure codes in Taiwan National Health Insurance (NHI) for Crohn’s Disease complications**

| NHI Code | Description |
| --- | --- |
| 73010B | Resection of small bowel, with anastomosis |
| 73011B | Colectomy, partial, with anastomosis |
| 73012B | Colectomy, radical hemicolectomy with anastomosis, ascending colon |
| 73013B | Left hemicolectomy or sigmoid colectomy |
| 73014B | Left hemicolectomy or sigmoid colectomy with anastomosis with lymph node |
| 73015B | Colectomy, total or subtotal - benign |
| 73017B | Colectomy, total with proctectomy, with ileostomy |
| 73022B | Enterostomy (including colostomy, Jujunostomy, permanent enterostomy) |
| 73032B | Anastomosis of bowel-for intestinal atresia or stenosis |
| 73033B | Repair of intestinal perforation |
| 73038B | Take down of anastomosis, revision of ileo-colostomy and reconstruction |
| 73042B | Revision of colostomy or enterostomy complicated, deep |
| 73044B | Laparoscopic jejunostomy |
| 73045B | Laparoscopic right colectomy and anastomosis |
| 73046B | Laparoscopic Anterior resection and anastomosis (sigmoid colon resection) - benign |
| 74205B | Radical protectomy with pelvic lymph node dissection |
| 74206B | Harmann operation - benign |
| 74212B | Rectoplasty for stricture or stenosis |
| 74222B | Proctosigmoidectomy with pull through colon anal anastomosis, reconstruction with colonic pouch |
| 75805B | Exploratory laparotomy |

**Supplementary Table S4. Duration of perianal Crohn’s disease episodes**

| Episode | Number of patients | Episode duration (day) |
| --- | --- | --- |
|  |  | Median (IQR) |
| First episode | 358 | 116 (81-181) |
| Second episode | 121 | 137 (88-229) |
| Third episode | 45 | 148 (110-257) |
| Forth episode | 19 | 130 (71-219) |
| Fifth episode | 10 | 142 (88-542) |
| Sixth (and more) episode | 12 | 270 (183-371) |

IQR: interquartile range.

**Supplementary Table S5.** **Temporal sequence between first diagnosis of Crohn’s disease and first diagnosis of perianal fistula among 256 patients in Taiwan from RCIPD dataset.**

| Diagnosis of perianal fistula from RCIPD | N=256 | Days between first Crohn’s disease diagnosis and first diagnosis of perianal disease | |
| --- | --- | --- | --- |
|  |  | Mean (SD) | Median (IQR) |
| More than 6 months before diagnosis of Crohn’s disease | 119 | 1,631 (1,95) | 1,310 (811-2,246) |
| Within 6 months before or after diagnosis of Crohn’s disease | 56 | 57 (48) | 30 (3-86) |
| More than 6 months after diagnosis of Crohn’s disease | 81 | 1,517 (1,212) | 1,350.5 (539-1,900) |

IQR: interquartile range

**Supplementary Table S6. Outcomes among 358 patients with perianal disease before or on/after diagnosis of Crohn’s disease during three years of follow-up**

|  | Perianal disease diagnosis was 6 months or more before Crohn’s disease diagnosis  N=170 | | Perianal disease diagnosis less than 6 months before or after Crohn’s disease diagnosis  N=188 | | *p*-value |
| --- | --- | --- | --- | --- | --- |
|  | N | Episodes  Mean (SD) | N | Episodes  Mean (SD) |  |
| Surgical intervention | 24 | 1.2 (0.5) | 55 | 1.1 (0.4) | 0.70 |
| Hospitalizations | 161 | 2.5 (2.5) | 160 | 3.7 (3.1) | <0.01 |
| Outpatient visits | 170 | 18.5 (18.6) | 188 | 23.1 (17.3) | 0.02 |
| Emergency visits | 107 | 2.9 (2.3) | 142 | 4.3 (3.8) | <0.01 |

**Supplementary Table S7. Baseline characteristics of 2,424 patients with Crohn’s disease in Taiwan.**

|  | **CD with Perianal Fistula** | |  | **CD without Perianal Fistula** | | **p-value** |
| --- | --- | --- | --- | --- | --- | --- |
|  | **N** | **(%)** |  | **N** | **(%)** |  |
| **Total** | 308 | (100.0) |  | 2116 | (100.0) |  |
| **Sex** |  |  |  |  |  | <0.01 |
| Male | 249 | (80.8) |  | 1283 | (60.6) |  |
| Female | 59 | (19.2) |  | 833 | (39.4) |  |
| **Age (CD)^a1^** |  |  |  |  |  |  |
| Mean (SD) | 33.0 | (14.3) |  | 44.8 | (21.7) | <0.01 |
| Median (Q1-Q3) | 31 | (22-42) |  | 45 | (28-62) |  |
| 0-19 | 44 | (14.3) |  | 282 | (13.3) | <0.01 |
| 20-39 | 173 | (56.2) |  | 597 | (28.2) |  |
| 40-59 | 78 | (25.3) |  | 625 | (29.5) |  |
| 60-79 | 13 | (4.2) |  | 524 | (24.8) |  |
| >=80 |  |  |  | 88 | (4.2) |  |
| **Age (Fistula)^a3^** |  |  |  |  | | |
| Mean (SD) | 32.5 | (14.9) |  |  |  |  |
| Median (Q1-Q3) | 28 | (21-43) |  |  |  |  |
| 0-19 | 50 | (16.2) |  |  |  |  |
| 20-39 | 163 | (52.9) |  |  |  |  |
| 40-59 | 77 | (25.0) |  |  |  |  |
| 60-79 | 18 | (5.8) |  |  |  |  |
| >=80 | 0 | 0 |  |  |  |  |
| **Comorbidity during a three-year baseline period ^e3^** | | | |  |  |  |
| Hypertension | 17 | (5.5) |  | 365 | (17.2) | <0.01 |
| Diabetes | 9 | (2.9) |  | 184 | (8.7) | <0.01 |
| Chronic kidney disease | 34 | | | | | 0.16 |
| Coronary artery disease | 7 | (2.3) |  | 144 | (6.8) | <0.01 |
| Liver disease | 33 | (10.7) |  | 241 | (11.4) | 0.77 |

**Supplementary Table S8. Drug use among 2,424 patients with Crohn’s disease in Taiwan**

|  | **CD with Perianal Fistula (N=308)** | | **CD without Perianal Fistula (N=2116)** | |  |
| --- | --- | --- | --- | --- | --- |
|  | **N** | **(%)** | **N** | **(%)** | **p-value** |
| **Medication** |  |  |  |  |  |
| 5-ASA | 289 | (93.8) | 1,459 | (69.0) | <0.01 |
| Steroid^b^ | 287 | (93.2) | 1,845 | (87.2) | <0.01 |
| Azathioprine | 193 | (62.7) | 657 | (31.0) | <0.01 |
| Anti-TNF-alpha | 141 | (45.8) | 334 | (15.8) | <0.01 |
| Vedolizumab | 6 | (1.9) | 4 | (0.2) | <0.01 |
| Antibiotics | 242 | (78.6) | 1,161 | (54.9) | <0.01 |
| Metronidazole | 226 | (73.4) | 947 | (44.8) | <0.01 |
| Ciprofloxacin | 140 | (45.5) | 599 | (28.3) | <0.01 |

**Supplementary Table S9. Temporal sequence between first diagnosis of Crohn’s disease and first diagnosis of perianal fistula among 308 patients in Taiwan**

| Diagnosis of perianal fistula | N=308 | Days between first Crohn’s disease diagnosis and first diagnosis of perianal fistula | |
| --- | --- | --- | --- |
|  |  | Mean (SD) | Median (IQR) |
| More than 6 months before diagnosis of Crohn’s disease | 141 | 1,611 (1,213) | 1,266 (756-2,237) |
| Within 6 months before or after diagnosis of Crohn’s disease | 65 | 54 (51) | 36 (3-92) |
| More than 6 months after diagnosis of Crohn’s disease | 102 | 1,651 (1,3222) | 1,292 (539-2,097) |

**Supplementary Table S10. Duration of intervals between episodes of perianal fistula among 308 Crohn’s disease patients**

| Recurrence interval | Number of episodes of second or subsequent perianal fistula | Days between intervals |
| --- | --- | --- |
|  |  | Median (IQR) |
| Total | 181 | 224 (57-829) |
| First recurrence | 102 | 236 (75-1,020) |
| Second recurrence | 39 | 270 (45-1,139) |
| Third recurrence | 19 | 143 (52-260) |
| Forth recurrence | 10 | 258 (148-521) |
| Fifth (and more) recurrence | 11 | 96 (31-381) |

IQR: interquartile range

**Supplementary Table S11. Outcomes among 2,424 Crohn’s disease patients with or without perianal fistula during ten years of follow-up**

|  | With perianal fistula  N=308 | | Without perianal fistula  N=2,116 | | | *p*-value |
| --- | --- | --- | --- | --- | --- | --- |
|  | N | Episodes  Mean (SD) | N | Episodes  Mean (SD) | |  |
| Surgical intervention | 116 | 1.3 (0.7) | 377 | 1.2 (0.5) | | 0.02 |
| Hospitalization | 301 | 5.9 (5.5) | 922 | 3.6 (4.3) | | <0.01 |
| Outpatient visits | 308 | 15.5 (13.5) | 2065 | 4.0 (3.4) | | <0.01 |
| Emergency visit | 277 | 7.3 (10.1) | 643 | 4.0 (6.0) | | <0.01 |
|  | N | % | N | % | |  |
| Colorectal cancer | 16 | | | | 1 | |
| Death | 11 | 3.6% | 285 | 13.5% | | <0.01 |
